# Imatinib treatment of poor prognosis mesenchymal-type primary colon cancer: a proof-of-concept study in the preoperative window period (ImPACCT)

**DOI:** 10.1186/s12885-017-3264-y

**Published:** 2017-04-19

**Authors:** I. Ubink, H. J. Bloemendal, S. G. Elias, M. A. Brink, M. P. Schwartz, Y. C. W. Holierhoek, P. M. Verheijen, A. W. Boerman, R. H. J. Mathijssen, W. W. J. de Leng, R. A. de Weger, W. M. U. van Grevenstein, M. Koopman, M. P. Lolkema, O. Kranenburg, I. H. M. Borel Rinkes

**Affiliations:** 1 Department of Surgical Oncology, Cancer Center, University Medical Center Utrecht, Heidelberglaan 100, 3584CX Utrecht, The Netherlands; 2 Department of Medical Oncology, Cancer Center, University Medical Center Utrecht, Heidelberglaan 100, 3584CX Utrecht, The Netherlands; 3 Department of Medical Oncology, Meander Medical Center, Maatweg 3, 3813TZ Amersfoort, the Netherlands; 4 Julius Center for Health Sciences and Primary Care, University Medical Center Utrecht, Universiteitsweg 100, 3584CG Utrecht, The Netherlands; 5 Department of Gastroenterology, Meander Medical Center, Maatweg 3, 3813TZ Amersfoort, the Netherlands; 6 Department of Surgery, Meander Medical Center, Maatweg 3, 3813TZ Amersfoort, the Netherlands; 7 Department of Medical Oncology, Erasmus MC Cancer Institute, ‘s-Gravendijkwal 230, 3015CE Rotterdam, the Netherlands; 8 Department of Pathology, University Medical Center Utrecht, Heidelberglaan 100, 3584CX Utrecht, The Netherlands

**Keywords:** Colon carcinoma, Targeted therapy, Imatinib, Proof-of-concept, Pre-operative window, RNA sequencing

## Abstract

**Background:**

The identification of four Consensus Molecular Subtypes (CMS1–4) of colorectal cancer forms a new paradigm for the design and evaluation of subtype-directed therapeutic strategies. The most aggressive subtype - CMS4 - has the highest chance of disease recurrence. Novel adjuvant therapies for patients with CMS4 tumours are therefore urgently needed. CMS4 tumours are characterized by expression of mesenchymal and stem-like genes. Previous pre-clinical work has shown that targeting Platelet-Derived Growth Factor Receptors (PDGFRs) and the related KIT receptor with imatinib is potentially effective against mesenchymal-type colon cancer. In the present study we aim to provide proof for the concept that imatinib can reduce the aggressive phenotype of primary CMS4 colon cancer.

**Methods:**

Tumour biopsies from patients with newly diagnosed stage I-III colon cancer will be analysed with a novel RT-qPCR test to pre-select patients with CMS4 tumours. Selected patients (*n* = 27) will receive treatment with imatinib (400 mg per day) starting two weeks prior to planned tumour resection. To assess treatment-induced changes in the aggressive CMS4 phenotype, RNA sequencing will be performed on pre- and post-treatment tissue samples.

**Discussion:**

The development of effective adjuvant therapy for primary colon cancer is hindered by multiple factors. First, new drugs that may have value in the prevention of (early) distant recurrence are almost always first tested in patients with heavily pre-treated metastatic disease. Second, measuring on-target drug effects and biological consequences in tumour tissue is not commonly a part of the study design. Third, due to the lack of patient selection tools, clinical trials in the adjuvant setting require large patient populations. Finally, the evaluation of recurrence-prevention requires a long-term follow-up. In the ImPACCT trial these issues are addressed by including newly diagnosed pre-selected patients with CMS4 tumours prior to primary tumour resection, rather than non-selected patients with late-stage disease. By making use of the pre-operative window period, the biological effect of imatinib treatment on CMS4 tumours can be rapidly assessed. Delivering proof-of-concept for drug action in early stage disease should form the basis for the design of future trials with subtype-targeted therapies in colon cancer patients.

**Trial registration:**

ClinicalTrials.gov: NCT02685046. Registration date: February 9, 2016.

## Background

Mortality from colon cancer is almost invariably due to the development of distant metastases. In current practice, pathological (TNM stage) and clinical characteristics (age, co-morbidity) mainly determine the choice of adjuvant chemotherapy. However, these features have limited value in predicting which patients are at risk of developing metastases. In clinical trials, the five-year recurrence rate in stage III colon cancer patients is approximately 50% without adjuvant chemotherapy. With adjuvant treatment this is reduced to ~35%, implying that such treatment is only effective in a subgroup of patients [1]. Consequently, the majority of patients are currently being under- or over-treated. It is therefore important to be able to identify patients who are at high risk of recurrence and to develop more effective therapies to prevent relapse. Relapse-prevention trials in the adjuvant setting are challenging however, due to the long follow-up periods and the large numbers of patients that are required for sufficient statistical power. Prior evidence of drug activity and the availability of a companion diagnostic tool for patient selection could greatly facilitate the design and increase the quality of such studies.

Novel adjuvant therapies should be based on an understanding of the pathways that drive metastasis formation. Recent studies on molecular classification of colon cancer have provided insight into these pathways. Several research groups have independently developed classification systems for primary colon cancer based on gene expression profiles [2–8]. Cross-cohort analysis of the results has led to the identification of four consensus molecular subtypes (CMS1–4). Of these, CMS4 (~25% of colon cancers) is associated with a significantly worse disease-free and overall survival [9]. Novel treatment strategies for this subtype are thus particularly needed.

The pro-metastatic pathways that are upregulated in CMS4 provide opportunities for subtype-targeted therapy. CMS4 tumours are characterised by high expression of stem cell and mesenchymal genes, and a high stromal content [9]. We have previously shown that Platelet-Derived Growth Factor Receptors (PDGFRs) and KIT are highly expressed in mesenchymal-type colon tumours and that their expression strongly correlates with disease-free survival. Moreover, in vitro and in vivo inhibition of PDGFR and KIT tyrosine kinase signalling reduced invasiveness, metastatic potential and stem-like characteristics of mesenchymal-type colon cancer [10–12]. Based on these findings we hypothesise that patients with poor-prognosis mesenchymal-type colon cancers could benefit from treatment with imatinib, a tyrosine kinase inhibitor with high selectivity for PDGFR and KIT.

To test this hypothesis in a proof-of-concept study, we designed the ImPACCT trial (*Im*atinib as *P*re-operative *A*nti-*C*olon *C*ancer *T*argeted therapy). In ImPACCT, patients with CMS4 colon cancer are identified with a recently developed 4-gene RT-qPCR test that measures *PDGFRA, PDGFRB, PDGFC* and *KIT* expression levels in diagnostic tumour biopsies [13]. Pre-selected patients with CMS4 tumours are then treated with imatinib during the pre-operative window period (the time between initial cancer diagnosis and surgery). This allows comparison of pre-treatment diagnostic tumour biopsies with biopsies obtained from the resection specimen after treatment. The primary objective is to assess whether imatinib treatment reduces the aggressive phenotype of CMS4 tumours in colon cancer patients. ImPACCT may not only form the basis for future adjuvant studies with imatinib, but could also serve as a blueprint for other proof-of-concept studies with subtype-targeted therapies.

## Methods

### Study design

**Fig. 1:**
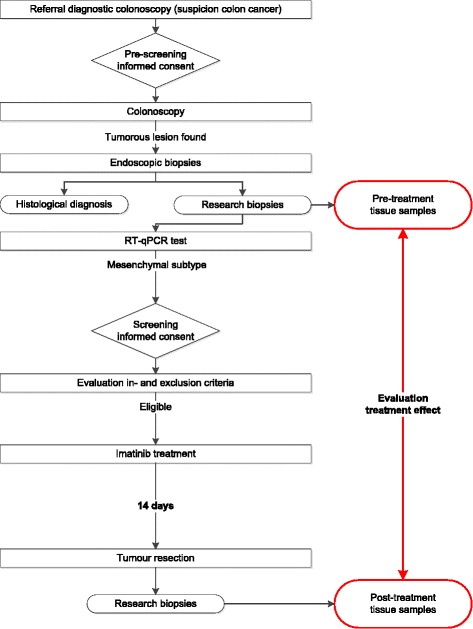
Study flow chart

The ImPACCT trial is an open-label, multi-centre proof-of-concept study. The primary endpoint of this trial is the effect of imatinib treatment on tumour biology, which is a pharmacodynamic endpoint, and as such this trial could be deemed a phase II/translational trial. A study flow chart is depicted in Fig. 1.

### Objectives

The primary objective of this trial is to investigate the effects of treatment on pro-metastatic pathways in aggressive primary CMS4 tumours. RNA sequencing will be performed on pre- and post-treatment tissue samples to document imatinib-induced genome-wide gene expression changes.

Secondary objectives include: to assess the extent of inhibition of PDGFR- and KIT after imatinib treatment; to relate intra-tumoural imatinib pharmacokinetics (PK) to systemic imatinib concentrations; to relate the level of inhibition of PDGFR/KIT signalling and the extent of changes in gene expression to the systemic and intra-tumoural PK of imatinib and its active metabolite CGP74588; to assess changes in tumour markers during treatment by measuring the concentrations of plasma-CEA and circulating tumour DNA (ctDNA) and to study the effects of imatinib on organoid-forming potential. Finally, the effect of short-term exposure to imatinib immediately followed by bowel surgery on the complication rate will be monitored.

### Study population

All patients who are scheduled for a diagnostic colonoscopy on account of clinical suspicion of colon carcinoma will be approached for permission to obtain extra biopsies for this study in case a tumour is found in the colon. These biopsies are pre-screened with the new RT-qPCR test that predicts the chance of a tumour being CMS4, based on the combined expression of imatinib targets *PDGFRA, PDGFRB, PDGFC* and *KIT* [13]. If the predicted chance of CMS4 in the biopsies is 50% or higher, patients will be approached for imatinib therapy. The study population that undergoes treatment with imatinib will thus consist of treatment-naïve newly diagnosed colon cancer patients with a tumour with a high probability of having the CMS4 phenotype. In- and exclusion criteria for enrolment in the second part of the trial (imatinib therapy) are listed in Table 1.

**Table 1:** In- and exclusion criteria for treatment with imatinib

Inclusion criteria	Exclusion criteria
- Male or female aged ≥18 years; - Histologically proven adenocarcinoma of the colon; - Completed cancer staging with CT-abdomen and CT-thorax/X-thorax according to hospital’s standard of care; - Confirmed eligibility for surgery with curative intent as deemed by the hospital’s multidisciplinary board review; - Test positive for CMS4 subtype; - ≥4 properly stored pre-treatment biopsies for gene expression analysis/ELISA; - WHO performance status 0 or 1; - Adequate haematology status and organ function (defined as: normal creatinine clearance (≥60 ml/min (MDRD)), ALAT within 2.5× upper limit of normal (ULN), PT-INR < 1.5, leukocytes >1,5*10^9/L, Hb > 6.0 mmol/L, platelets >100*10^9/L); - Willingness and ability to comply with scheduled visits, treatment plans and laboratory tests; - Provision of written informed consent.	- The presence of synchronous distant metastases; - Current hospital standard of care dictates that subject should undergo any neoadjuvant therapy; - Concurrent participation in another clinical trial using any medicinal product, or participation in such a trial in the period of three months prior to the current trial; - Women who are pregnant, plan to become pregnant or are lactating during the study or for up to 30 days after the last dose of imatinib; - Known HIV or Hepatitis B/C infection; - Known symptomatic congestive heart failure; - Co-morbidity requiring concomitant treatment with drugs that act as strong inducers of CYP3A4 or with drugs with a narrow therapeutic range influenced by imatinib.

### Study procedures

Patients pre-selected with the RT-qPCR test will be screened by a medical oncologist for inclusion in the second part of this study. Included patients will receive treatment with imatinib starting two weeks prior to planned tumour resection. Imatinib will be administered orally at a daily dosage of 400 mg for two weeks, with the last dose given within 12 h before surgery. Patients are requested to register drug intake and any adverse events in a patient diary. Before the start of imatinib therapy and at the end of the treatment period, blood samples will be obtained to measure ctDNA and plasma-CEA. Plasma imatinib trough levels will be determined on the day of surgery. Immediately following tumour resection, biopsies will be taken from the surgical specimen (post-treatment biopsies). Gene and protein expression of the pre-treatment biopsies (from colonoscopy) and post-treatments biopsies will be compared to assess the effects of imatinib therapy on PDGFR- and KIT-signalling and on the mesenchymal gene expression profile. After surgery, patients will be monitored according to standard of care. Any postoperative adverse events up until 14 days after discontinuation of study medication (end of study) will be documented.

### Sample size calculation

This study is designed as a proof-of-concept study with multiple outcomes of interest. We expect the effect size of imatinib treatment on the various parameters to be very high, since we specifically select patients who express high levels of the drug targets. However, we are aware of possible factors that may reduce the observed effect size. This includes intra-tumour heterogeneity in target expression – causing potential misclassification – and variation in drug distribution throughout the tumour and between patients. Therefore, we anticipate a medium to high Cohen effect size for the primary endpoint (i.e. Cohen’s effect size of 0.65). To demonstrate such an effect, we need to include 27 (evaluable) patients, based on a two-sided paired-samples t-test with a significance level α of 0.05 and power 1-β of 90%. We specifically chose this high power in order not to dismiss effects that are potentially relevant for further development of imatinib therapy in colorectal cancer patients. Given that approximately 25% of colon cancers are CMS4, at least 4*27 = 108 eligible patients with newly diagnosed colon cancer will need to be pre-screened with the RT-qPCR test. Since <10% of the patients undergoing colonoscopy are diagnosed with colon cancer, at least 1.100 patients will have to be approached for pre-screening informed consent.

### Statistical analyses

Imatinib-induced changes in the expression of specific gene signatures associated with CMS4 will be assessed by RNA-sequencing analysis. The signatures will be obtained from published literature and our own data. In addition, imatinib-induced gene expression changes will be analysed in a non-biased way by gene ontology and gene enrichment analyses. Using the appropriate statistical tests depending on distribution of the data (paired samples t-test in case of normal distribution or Wilcoxon signed-rank test if the distribution remains non-normal even after transformation) we will test whether there is a significant change in gene signature expression. Phosphorylation of PDGFRα/β and KIT will be quantitatively assessed as a fraction of the total amount of PDGFRα/β and KIT present in a sample. Post-treatment imatinib-induced inhibition of PDGFR phosphorylation will be compared relative to the pre-treatment sample (i.e. the patient’s internal control) using the appropriate statistical tests. Correlations between plasma trough levels and intratumoural concentration of imatinib, and between the extent of PDGFR and KIT inhibition and systemic/intratumoural imatinib concentrations will be evaluated using Pearson correlation coefficient. The Wilcoxon matched pairs signed rank test will be used to compare serial ctDNA and CEA levels. Organoid-forming potential of the tumours after imatinib therapy will be compared with the general success rate of organoid-establishment from colon cancers, using a Fisher exact test. In case of missing data, samples will be excluded pair-wise.

## Discussion

Targeted anti-cancer therapies that seem promising in pre-clinical and early-phase clinical trials often fail to show benefit in phase III randomized controlled trials. Up to 60 % of phase III trials have negative outcomes [14, 15]. This high failure rate underscores the need for optimization of trial design, including better patient selection. Since the successful addition of oxaliplatin to the adjuvant chemotherapy regimen in 2004 [16], no further advances have been made in the outcome of patients with stage II/III colon cancer. The lack of recent positive adjuvant chemotherapy trials is partly due to the fact that hardly any new drugs are actually tested in the adjuvant setting. Novel treatments are generally first tested in patients with metastatic disease who have no regular treatment options left, and who have often received multiple lines of systemic treatment. Therapies aimed at preventing the development or outgrowth of metastases are probably most effective at early disease stages, but their clinical development may be terminated due to lack of efficacy in late-stage disease [17]. Moreover, new (combination) treatments that are effective in metastatic disease do not necessarily have value in the adjuvant setting, as exemplified by trials PETACC-03 (5-FU/LV plus irinotecan) [18], NSABP C-08 (FOLFOX6 plus bevacizumab) [19] and N0147 (FOLFIRI plus cetuximab) [20]. To address this problem, design and approval of clinical trials in which promising drugs are tested in treatment-naïve patients with early-stage disease, rather than in late-stage patients who progressed under standard treatment, is needed.

Prospective stratification and/or inclusion based on predictive molecular biomarkers will presumably improve trial results by enrichment for responsive patients [21]. However, microsatellite instability is currently the only molecular marker that is being used in the clinical decision process for adjuvant chemotherapy in colon cancer [22]. The four recently identified CMSs in colon cancer show marked differences in the activity of various biological pathways, which could provide the basis for subtype-specific targeted therapy [9]. CMS4 (the mesenchymal/stem-like/stroma-rich subtype) has the poorest prognosis and, importantly, this subtype seems associated with a lack of benefit from oxaliplatin treatment [23]. These findings call for clinical trials with novel therapies specifically targeting CMS4 tumours. In ImPACCT we aim to deliver proof-of-concept that PDGFR/KIT inhibition with imatinib reduces the aggressive phenotype of newly diagnosed primary CMS4 colon tumours. Delivering proof-of-concept for drug activity in early stages of drug development is pivotal to prevent unfounded trial phase transition [14, 15].

ImPACCT is conducted with treatment-naïve patients during the pre-operative window period, which allows us to obtain high quality tissue material before and after imatinib therapy without additional interventions: pre-treatment samples will be obtained during diagnostic colonoscopy and post-treatment biopsies are collected from the resection specimen; both procedures are part of standard of care. By comparison of pre- and post-treatment tissue samples, the effects of imatinib on CMS4 tumours can be evaluated at a cellular level. The treatment period of 14 days is within the normal time frame from diagnosis to surgery (the pre-operative window). We expect that two weeks of treatment will be sufficient to induce changes in gene expression: given that steady state plasma concentrations will be attained within four days (the plasma half-life of imatinib is 18 h [24]), patients will be exposed to the full dose for ten days. The chosen daily dose of 400 mg is the standard dose for chronic myeloid leukaemia, gastrointestinal stromal tumours (GIST) and myelodysplastic syndrome.

Experimental therapy during the pre-operative window period could potentially lead to an increase in postoperative complications. However, based on two phase II trials that evaluated the use of neoadjuvant imatinib therapy for otherwise irresectable or metastatic GIST, we believe it is safe to administer the last dose of imatinib within 12 h before surgery. In these trials imatinib was administered for several months and stopped one day prior to surgery. Both studies concluded that this approach was feasible, and the reported postoperative complications were acceptable and not out of the ordinary considering the extensive abdominal surgery [25, 26].

Instead of assessing a clinical endpoint (e.g. disease-free survival), this proof-of-concept trial investigates a surrogate endpoint (gene expression changes) to demonstrate a ‘biological’ treatment effect. This endpoint requires a relatively limited sample size and can be determined after only two weeks of therapy, which limits the burden placed on participants. If treatment effects are indeed demonstrated, knowledge of the size of the effect on tumour biology can be used to drive the design of a larger randomised phase II trial.

The design of the ImPACCT trial presented here allows rapid evaluation of the mechanism of action of a targeted therapy in a subtype-stratified patient population by analysis of paired pre- and post-treatment biopsies, without interfering with or delaying standard-of-care, by making use of the pre-operative window period. This design can serve as a blueprint for subtype-directed proof-of-concept trials in colon cancer, with the ultimate goal of designing effective adjuvant therapy that eradicates occult metastases.

## Abbreviations

ALAT: Alanine transaminase
CEA: Carcino-embryonic antigen
CMS: Consensus molecular subtype
ctDNA: Circulating tumour DNA
ELISA: Enzyme Linked Immuno-Sorbent Assay
GIST: Gastrointestinal stromal tumours
HIV: Human immunodeficiency virus
ImPACCT: Imatinib as Pre-operative Anti-Colon Cancer Targeted therapy
MDRD: Modification of Diet in Renal Disease
PDGF(R): Platelet-Derived Growth Factor (Receptor)
PK: Pharmacokinetics
PT-INR: Protrombine time - international normalized ratio
RT-qPCR: Reverse Transcriptase quantitative Polymerase Chain Reaction
WHO: World Health Organisation
